# Nanocarriers for Delivery of Anticancer Drugs: Current Developments, Challenges, and Perspectives

**DOI:** 10.3390/pharmaceutics16121527

**Published:** 2024-11-27

**Authors:** Umme Hani, Vikram T. Choudhary, Mohammed Ghazwani, Yahia Alghazwani, Riyaz Ali M. Osmani, Gururaj S. Kulkarni, Hosakote G. Shivakumar, Shahid Ud Din Wani, Sathishbabu Paranthaman

**Affiliations:** 1 Department of Pharmaceutics, College of Pharmacy, King Khalid University, Abha 61421, Saudi Arabia; uahmed@kku.edu.sa (U.H.); myghazwani@kku.edu.sa (M.G.); 2 Department of Pharmaceutics, The Oxford College of Pharmacy, Hongsandra, Bengaluru 560068, India; skguru2006@gmail.com; 3 Department of Pharmacology, College of Pharmacy, King Khalid University, Abha 61421, Saudi Arabia; ysghazwani@kku.edu.sa; 4 Department of Pharmaceutics, JSS College of Pharmacy, JSS Academy of Higher Education and Research (JSS AHER), Mysuru 570015, India; riyazosmani@jssuni.edu.in (R.A.M.O.); hgshivakumar@jssuni.edu.sa (H.G.S.); 5 Department of Pharmaceutical Sciences, School of Applied Sciences and Technology, University of Kashmir, Srinagar 190006, India; shahidpharma2013@gmail.com; 6 Department of Cell Biology and Molecular Genetics, Sri Devraj Urs Medical College, Sri Devaraj Urs Academy of Higher Education and Research, Kolar 563103, India; sathishbabu.p94@gmail.com

**Keywords:** cancer, drug delivery, nanomedicine, tumor metabolism, mRNA therapy

## Abstract

Cancer, the most common condition worldwide, ranks second in terms of the number of human deaths, surpassing cardiovascular diseases. Uncontrolled cell multiplication and resistance to cell death are the traditional features of cancer. The myriad of treatment options include surgery, chemotherapy, radiotherapy, and immunotherapy to treat this disease. Conventional chemotherapy drug delivery suffers from issues such as the risk of damage to benign cells, which can cause toxicity, and a few tumor cells withstand apoptosis, thereby increasing the likelihood of developing tolerance. The side effects of cancer chemotherapy are often more pronounced than its benefits. Regarding drugs used in cancer chemotherapy, their bioavailability and stability in the tumor microenvironment are the most important issues that need immediate addressing. Hence, an effective and reliable drug delivery system through which both rapid and precise targeting of treatment can be achieved is urgently needed. In this work, we discuss the development of various nanobased carriers in the advancement of cancer therapy—their properties, the potential of polymers for drug delivery, and recent advances in formulations. Additionally, we discuss the use of tumor metabolism-rewriting nanomedicines in strengthening antitumor immune responses and mRNA-based nanotherapeutics in inhibiting tumor progression. We also examine several issues, such as nanotoxicological studies, including their distribution, pharmacokinetics, and toxicology. Although significant attention is being given to nanotechnology, equal attention is needed in laboratories that produce nanomedicines so that they can record themselves in clinical trials. Furthermore, these medicines in clinical trials display overwhelming results with reduced side effects, as well as their ability to modify the dose of the drug.

## 1. Introduction

Cancer has been the second leading cause of death after cardiovascular diseases, and the number of cases continues to increase globally. Treatment options include surgery, chemotherapy, and radiotherapy. Among these, approximately 45% of cancers can be cured by surgery, while chemotherapy and radiotherapy can be used to treat a mere 5% of cancer patients [1]. The remaining cancer patients fail to recover. Conventional chemotherapy strategies have several issues, such as early disease detection, the development of multidrug resistance, toxicity and side effects, and poor treatment outcomes [2,3]. Additionally, the expected clinical outcomes associated with traditional nanocarriers often differ from those associated with anticancer therapy. Many existing antitumor drugs suffer from nonideal pharmaceutical and pharmacological properties, such as drug insolubility, irritant properties, rapid metabolism, nonspecificity, extensive biological distribution, short half-life, and toxic side effects, which can lead to suboptimal therapeutic activity with poor patient compliance, thereby eventually affecting the life of the patient [4].

The concept of nanotechnology was first proposed by Richard P. Feynman in 1965. Nanotechnology is one of the eminent technologies to be followed by formulation researchers in the 21st century. It is used in diverse fields, such as physics, electronics, engineering, biology, agriculture, and medicine. Nanotechnology has beneficial effects in medicine and has made significant contributions to oncology. Nanomaterials are man-made, and such materials exhibit exceptionally high surface area-to-volume ratios, resulting in diverse physical and chemical properties and functions, with one external dimension that measures 100 nanometers (1 nm = 10^−9^ m) [5,6]. Owing to the specifications of atoms and molecules in nanomaterials, they possess the unique ability to control and manipulate systems. Nanomaterials include nano-objects, e.g., nanoparticles, nanofibers, nanotubes, nanoshells, and nanoplates, where the active pharmaceutical ingredient can be encapsulated, entrapped, dissolved, or attached to the drug carrier. They are made up of different materials and usually exist as agglomerates and aggregates [7,8]. Nanomedicine helps in the exploration of deeper cells and tissues and helps fight various diseases, including monster diseases such as cancer. Nanomedicines are commonly used to improve both the efficacy and toxicity of chemotherapeutic drugs by modulating their biodistribution and target site accumulation.

**Figure 1:**
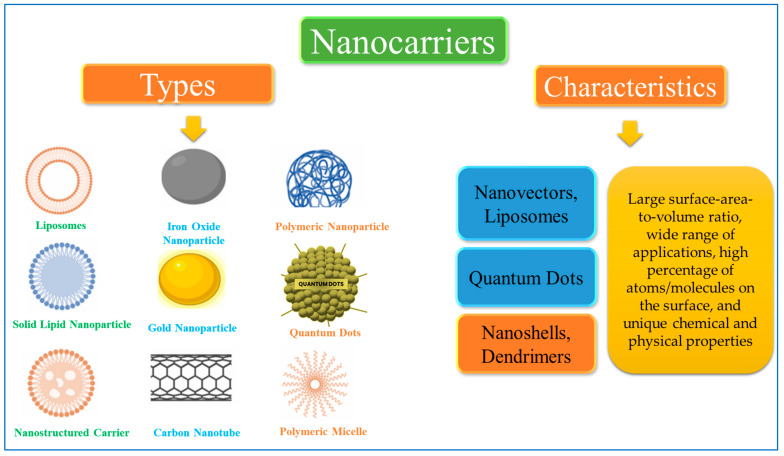
Various types of nanocarriers used in cancer treatment.

Nanocarriers used as vehicles can overcome cancer complications and improve therapeutic outcomes by delivering drugs in a controlled manner to specific target sites [9,10]. Furthermore, they can reduce toxicity and increase drug efficacy, thereby improving the drug therapeutic index [11]. Additionally, steady-state drug levels can be achieved over an extended period. The most commonly used and trending nanocarriers include nanovectors, liposomes, dendrimers, nanoshells, quantum dots, etc., through which anticancer drugs can be delivered to the target site. Figure 1 shows various types of nanocarriers used in cancer treatment. Nanotechnology is a rapidly growing field that is evolving and improving continuously and exerting prominent effects on cancer.

The therapeutic efficacy of drugs from various therapeutic groups, including anticancer drugs, can be effectively improved via the proper selection of polymers, the selection of suitable delivery vehicles, and the subsequent loading of the vehicles into suitable dosage forms as drug delivery systems [12,13]. They act as pharmacological carriers. In the drug delivery process, a pharmaceutical compound is administered to elicit a therapeutic effect. Different nanobased drug delivery systems include nanoemulsions, nanotubes, nanorods, nanowires, micelles, nanoparticles, nanofibers, etc. These formulations possess special abilities that entrap, retain, transport, and eventually deliver drug molecules to target sites, and this has drawn much attention in the medical field [11,14].

Nanobased drug delivery systems are thus potential candidates through which drugs can be entrapped or retained in nanocarriers. Thus, drug-entrapped nanocarriers act as efficient vehicles through which drugs can be effectively delivered in the vicinity of target sites in various diseases, including cancer. Additionally, nanobased drug delivery systems can circumvent drug-related problems such as side effects and aid in improving the therapeutic efficacy of pharmaceutical compounds [11,14,15]. Polymeric delivery systems are also used as drug delivery systems. Both natural and synthetic polymers can entrap diverse biomolecules and provide various therapeutic benefits, such as the controlled release of drugs, protection of drugs from premature degradation, and reduced drug toxicity [16,17]. Drugs able to be delivered by polymers bind to the outer layer, thereby offering increased solubility and stability [18].

Natural and synthetic polymers are used for nanoencapsulation. Natural polymers include starch, gelatin, pectin, cellulose, alginate, albumin, chitosan (CS), etc., while synthetic polymers such as polyacrylates, polymethylacrylates, and polycaprolactone are used. Natural polymers possess special properties, such as nontoxicity, nonreactivity, biodegradability, and biocompatibility, and are thus preferred over synthetic polymers [19,20]. Despite the availability of several immunological treatments for cancer, the results remain unsatisfactory in cancer patients. This could be attributed to the tumor metabolic microenvironment (TMME), which inhibits a variety of antitumor immune responses, thereby reducing the effectiveness of cancer immunotherapies. Furthermore, owing to their ability to upregulate tumor-suppressing proteins and inhibit metastases, the use of mRNA-based nanotherapy in cancer has garnered much attention. The present review discusses various nanotechnology advances in cancer treatment and their properties, biopolymers and drug delivery potential, issues in anticancer drug delivery, advances in formulations, and key issues such as nanoparticle distribution and the toxicity of in vivo imaging. Additionally, we discuss the most recent advancements in the engineering of nanomedicines that enhance antitumor immunity by regulating the metabolism of glucose and amino acids, as well as mRNA-based nanotherapeutics that restore the expression of tumor-suppressor genes and suppress the expression of oncogenes.

## 2. Nanovectors

Nanovectors or nanocarriers are nanoscale components that aid in delivering therapeutic molecules or contrast agents to a specific location at the proper concentration and time [21]. These nanoparticles are highly important in cancer therapy and are used as anticancer and detection agents. They are classified into three generations.

First-generation nanovectors resemble current clinical liposomes. They consist of an exterior phospholipid bilayer, and the active principle forms the interior cavity. Examples of first-generation nanovectors include albumin-bound paclitaxel nanoparticles prescribed for breast cancer chemotherapy. In this case, with the aid of the enhanced permeation and retention (EPR) mechanism, the drug is localized within the tumor tissue. This localization mechanism results in an altered pharmacokinetic profile, including bioavailability and toxicity of the active principle [21]. The main limitation of first-generation nanovectors is their inability to target specific biomolecules in tumor cells.

Second-generation nanovectors are the result of the gradual evolution of first-generation nanovectors. These act by targeting specific biomolecules in tumor cells. These systems consist of delivery systems with additional functionality and surface moieties such as antibodies, ligands, aptamers, and small peptides. These surface moieties on nanovectors provide molecular recognition of receptors over tumor cells or within tumor cells and provide active or triggered drug release at diseased locations [21]. Compared with first-generation nanovectors, second-generation nanovectors show better biodistribution and reduced toxicity. Examples include antibody-targeted nanoparticles such as mAb-conjugated liposomes [22,23,24]. Numerous targeting moieties, such as ligands, aptamers, peptides, and phage-display peptides, are under investigation worldwide, in addition to antibodies [25,26].

**Figure 2:**
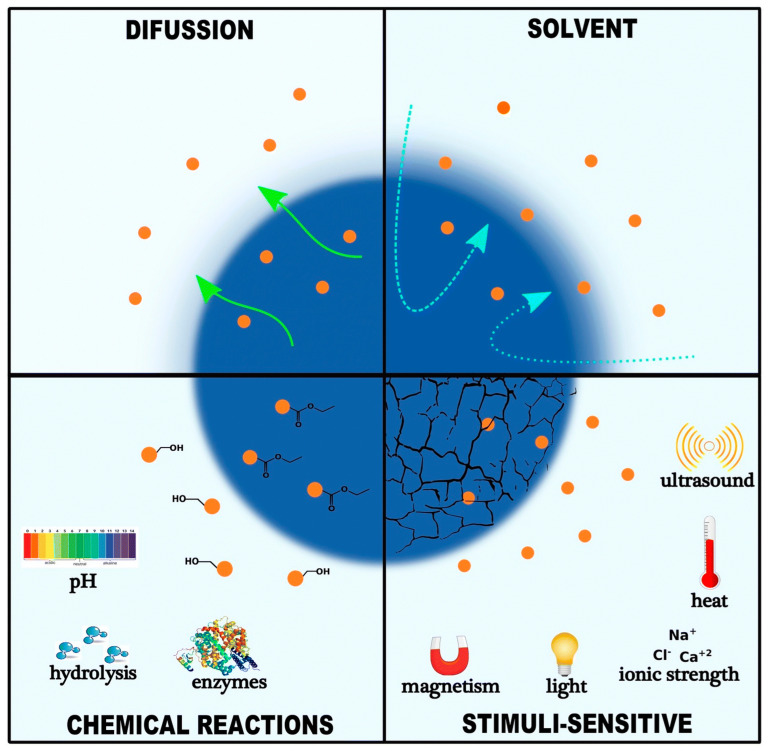
Techniques for regulating medication release via various types of nanocarriers. Reproduced with permission from [28], which was published under a CC BY license.

Third-generation nanovectors are multistage agents capable of multiple tasks, such as providing time-controlled release of pharmaceutical compounds from nanoparticles, overcoming biological barriers, and generating distinct subcellular targets [27]. Many studies have reported the release processes of medications within nanocarriers. Several processes, including solvent diffusion, chemical reactions, and stimuli-controlled release, can be used to describe how medications are released from nanocarriers, as shown in Figure 2 [27,28].

This current generation of nanovectors is designed to address biobarrier issues and to improve or enhance drug delivery to the tumor location. Third-generation nanovectors have been developed to circumvent the challenges of chemotherapeutic strategies. Examples include ‘nanoshuttles’ and ‘nanocells’ [29,30].

Albumin is a tiny, globular protein produced by the liver [31] and accounts for approximately 55% of blood proteins. It performs several functions, such as maintaining colloid osmotic pressure and regulating the plasma pH [32]. Furthermore, it is involved in controlling the transport of nutrients [33], metal ions [34] and systemic drugs, thereby enhancing both drug bioavailability and stability in the biological milieu [31]. For these reasons, albumin can be conjugated with active principles, resulting in enhanced pharmacokinetic properties of the drug [35]. Therefore, albumin was fabricated for the development of safe and cost-effective nanovectors [36].

Fenretinide, a synthetic retinoid derivative, has an excellent pharmacological profile; however, its clinical use is limited due to its low bioavailability. Nanoencapsulation of proteins and peptides protects the active principles against degradation while also modifying or extending drug release, resulting in the maintenance of effective drug plasma concentrations for a prolonged time period. Albumin has a solubilization effect on hydrophobic drugs and is known to improve bioavailability. Fenretinide albumin nanocapsules displayed profound in vivo anti-proliferative effects and were able to kill 3D spheroids of lung cancer cells. Albumin nanocapsules were designed to improve treatments for non-small cell lung cancer (NSCLC) [37]. G. Battogtokh et al. (2015) developed paclitaxel-loaded cholesteryl albumin nanoparticles (PTX-Chol-BSA). The resulting nanoparticles displayed increased stability of drug-paclitaxel in the blood environment due to conjugation with cholesterol and high drug encapsulation efficiency. Upon the administration of PTX-Chol-BSA, pharmacokinetic studies in tumor-bearing mice revealed that the area under the concentration–time curve (AUC_0–8h_) was 1.6–2 times greater than that of PTX-BSA, indicating the presence of the drug in the plasma [38].

## 3. Liposomes

**Figure 3:**
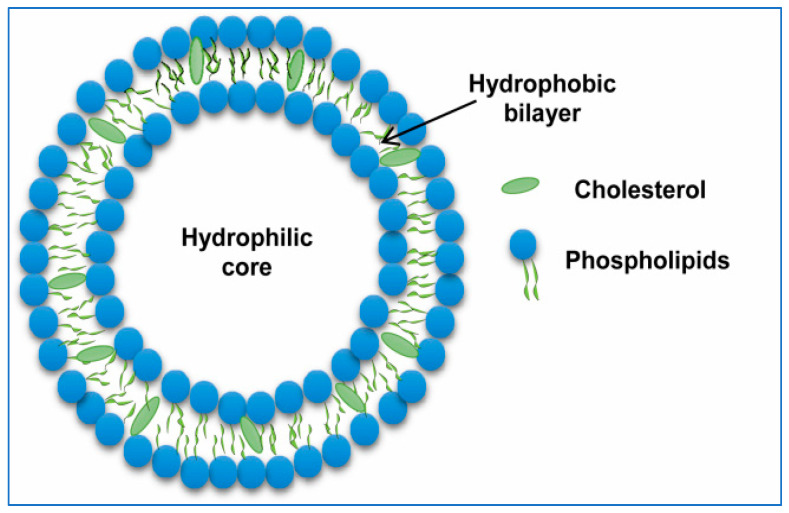
Schematic illustration of liposomes. Reproduced with permission from [39], which was published under a CC BY license.

Among lipid-based drug delivery systems, liposomes are widely used in nanomedicine. They are self-assembling, closed colloidal vesicles with a bilayered lipid layer surrounding the central aqueous core (Figure 3).

Dr. Alec. D Bangham et al. introduced ‘phospholipid spherules’ in the mid-1960s, which were first utilized as model membranes for the study of biological barriers [40]. However, the term ‘liposomes’ was discovered by Sessa and Weissman in 1968 [41]. Liposomes possess special characteristics, such as biodegradability, biocompatibility, nontoxicity, availability of various sizes, and ability to serve as solubilizing vehicles for drugs with poor aqueous solubility. Phosphatidylcholines, phosphatidylethanolamines, phosphatidylserines, sphingolipids, glycolipids, and sterols are some of the common phospholipids utilized in liposome synthesis.

**Figure 4:**
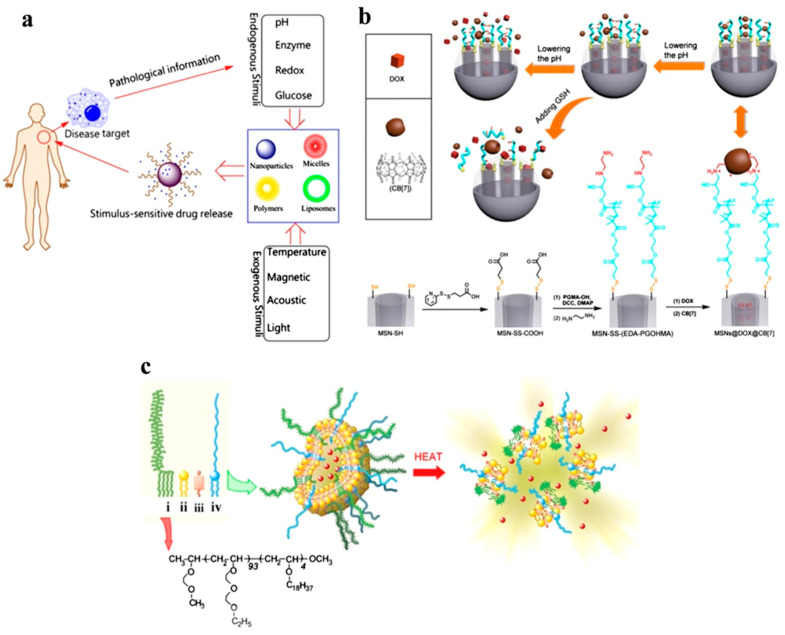
The stimuli-responsive DDS is illustrated schematically (**a**); the dual-responsive dynamic crosslinked supramolecular network on MSN-SS-(EDA-PGOHMA) and the synthetic approach with CB7 assembly are schematically illustrated in (**b**); and the temperature-sensitive liposome design is illustrated schematically in (**c**). Reproduced with permission from [48], which was published under a CC BY license.

Since liposomes possess the dual advantages of entrapping hydrophilic and hydrophobic drugs in the aqueous center and liposomal wall of the liposome, respectively, they are extensively researched nanocarriers. Drugs able to bind to liposomes, both into or onto their membranes, are transported to the site of action without the risk of degradation and minimum side effects since liposomes are made up of lipids that are inert, biodegradable and nonimmunogenic in nature. Additionally, lipids have a relatively low toxicity profile and do not elicit any antigenic or pyrogenic reactions [42,43,44]. Owing to these properties, surface modification through the targeting of ligands, such as proteins, peptides, antibodies, DNA/RNAs, and various other molecules, to achieve ideal characteristics makes liposomes excellent candidates for drug delivery [45,46]. Chemotherapeutic and diagnostic agents can be encapsulated in the inner aqueous core of liposomes or conjugated to the surface of liposomes [47]. Therapeutic chemicals are delivered to specific targets on the basis of factors that respond to exogenous stimuli (such as temperature, sound, or light) or endogenous stimuli (such as pH, redox, or enzymes), as shown in Figure 4. Insulin is administered orally via a supramolecular gel based on pH-sensitive chitosan. Because of the nanogel’s sensitivity to pH, insulin is protected while it is in the stomach, and the bioadhesivity of chitosan allows for extended contact with the intestinal mucosae, which increases insulin absorption. Doxorubicin, a chemotherapeutic anticancer agent, is delivered via vesicle pH-sensitive reactions, which increase the cytotoxicity of the drug to both drug-sensitive and drug-resistant cells [47,48].

**Figure 5:**
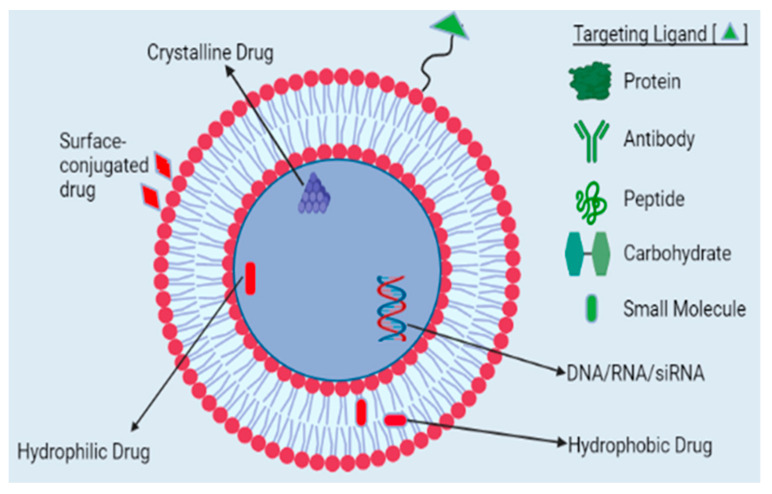
Liposomal drug delivery (created with BioRender.com, accessed on 25 August 2022).

They are the most versatile and nanobased drug delivery platforms for a diverse range of bioactive molecules [49]. Figure 5 depicts liposomal drug delivery. Liposome-based formulations have been shown to improve both drug pharmacodynamic and pharmacokinetic properties [50]. Liposomal amphotericin-B (Ambisome^®^) was the first approved product for fungal infections in 1990. Liposomal anthracyclines such as pegylated liposomal doxorubicin (Doxil), nonpegylated liposomal doxorubicin (Myocet), and daunorubicin citrate liposomes (DaunoXome) have been approved and are indicated for the treatment of metastatic breast cancer and Kaposi’s sarcoma [49,51,52,53].

The main aim of liposomal formulations is to minimize toxicity without affecting drug efficacy. These can be achieved via two strategies. First, through site avoidance, the encapsulated drug is impeded from arriving at healthy tissue, and second, through drug targeting, drug concentrations are aimed at delivery to neoplastic tissue [40].

Liposome uptake by the reticuloendothelial system (RES) results in fast clearance of liposomes from the blood, thereby impacting the distribution of liposomes and the active principles connected to tumors or the sites where they act [54]. To avoid rapid clearance of liposomes, surface-modified liposomes (stealth) (S-Liposomes) have been developed. Surface-modified liposomes consist of hydrophilic polymers, which are usually lipid derivatives of polyethylene glycol (PEG) grafted to the liposomal surface [50,55]. They offer several benefits over conventional liposomes, viz., longer circulation, preferential accumulation, enhanced uptake, and reduced toxicity. Thus, surface modification of liposomes addresses the issue of rapid clearance of liposomes from the blood stream, resulting in liposomes with an extended half-life [54].

Song et al. (2022) developed a liposomal drug delivery system with the inclusion of DSPE-MPEG 2000 (DP), a stabilizer to improve the poor aqueous solubility, low bioavailability, and stability of curcumin (CUR) and tetrandrine (TET). The resulting CT-DP liposomes displayed a stable particle size of 110 nm under different conditions, high solubility, and high encapsulation efficiency. Additionally, CT-DP liposomes have shown strong antitumor efficacy in a variety of cancer cells [56]. Miao et al. (2021) prepared paclitaxel-loaded CS-oligosaccharide (PTX-CSO) liposomes through the thin film hydration method for treating lung cancer. The CSO-modified liposomes were able to reduce the leakage of encapsulated paclitaxel in body fluids, and further, intravenous injection of paclitaxel-loaded CP50-loaded liposomes in an A549 tumor-bearing mouse model resulted in optimal anticancer therapeutic performance, with an excellent tumor inhibition rate of 86.4% [57].

Cellulose is considered a biological molecule with low toxicity, biocompatibility, and inert nature. It possesses special physical and chemical properties and is involved in transporting drugs to target sites; thus, it is considered ideal for a variety of applications in the medical field. In its original form, it is insoluble in water; hence, to address this problem, different forms of modified cellulose have been fabricated. Modified forms of cellulose, including cellulose ethers, methylcellulose, and hydroxypropyl cellulose, are used in nanobased drug delivery systems for numerous diseases, including cancer. Thus, the reconfigured forms of cellulose act as thickening and stabilizing agents, which further aids in the development of nanocarriers for the delivery of specific drugs [9].

Patel et al. (2020) synthesized phycobiliprotein (PBP) liposomes through the thin-film hydration method. Furthermore, PBP liposomes were stabilized through PEG adsorbed with cellulose nanocrystals (CNCs). Plant-based protein-loaded liposomes displayed controlled release of protein in the gastrointestinal milieu, thus indicating the stability of CNCs as coating materials and their potential for nutraceutical and food applications [58]. In another study, hydroxyethyl cellulose was used as a carrier for methotrexate. Compared with I.V. injection, free drug injection in in vivo orthotopically implanted mammary tumors resulted in high antitumor activities of 11.2% and 48.4%, respectively [59].

Pectin, another naturally occurring biopolymer, consists of α-(1–4)-d-galacturonic acid units. It has low solublity because of its indigestible nature in the GI tract. The pectinolytic enzymes generated by bacteria help absorb pectin in the colon [60]. Owing to its hydrophobic nature, it is used as a nanocarrier in the development of transdermal formulations of a few drugs. Pectin-based nanocarriers have been synthesized and formulated in the form of nanoparticles, nanoemulsions, and nanoliposome systems for both drug delivery and the treatment of diseases, including cancer. Numerous drugs, including cefazolin [61], metformin [62], and curcumin [63], with enhanced drug loading and encapsulation efficiency have been developed as nanocarriers of pectin.

**Figure 6:**
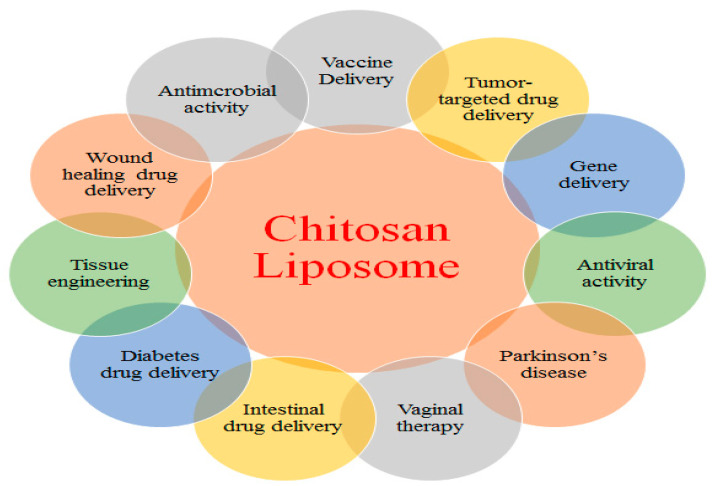
Multifaceted biomedical applications of CS-liposome-based systems. Reproduced with permission from [67], which was published under a CC BY license.

Chitosan, a linear polycationic polysaccharide polymer, is widely used for its multifaceted properties. It is the second most abundant biopolymer, after only cellulose. It is extracted from the exoskeleton of crustaceans and is widely used for a variety of pharmaceutical applications, such as drug delivery systems based on nanotechnology. It is a copolymer of β-1,4-linked 2-amino-2-deoxy-glucopyranose and 2-acetamido-2-deoxy residues-β-d-glucopyranose. It displays a broad range of biological characteristics, such as antitumor, antifungal, antimicrobial, antioxidant, and wound healing activities. Its excellent qualities, viz., nontoxicity, biocompatibility, biodegradability, and low cost, make it a useful element in nanobased drug delivery systems [9]. Additionally, its high serum stability, minimal immunogenicity, extended circulation period, and high bioavailability enable it to serve as an efficient nanocarrier [64,65,66]. Figure 6 shows various biomedical applications of CS-liposome-based systems [67].

W. Wang et al. developed CS-based thermosensitive hydrogels containing doxorubicin liposomes via the remote-loading method for the treatment of topical cancer. The hydrogel displayed excellent fluidity, strong anticancer activity, and decreased systemic toxicity at room temperature [68]. In another study, Kaldybekov et al. prepared mucoadhesive maleimide-functionalized PEGylated liposomes (PEG-Mal) for the release of vesicular drugs into the urinary bladder. They reported that PEGylated liposomes had a longer retention period than other liposomes did [69]. In one study, curcumin-loaded liposomes modified with CS resulted in the formation of positively charged, oligolamellar, and stiff vesicles. Furthermore, CS was able to protect herbal drugs and vesicles while also improving the local treatment of lung disease [70]. Avachat et al. developed a novel dressing of lyophilized CS-based liposomal wafers encapsulated with gemifloxacin for excision wounds. The developed liposomal wafers displayed good antibacterial activity against Gram-negative and Gram-positive bacteria in vitro. Furthermore, the application of a drug-loaded liposomal wafer in an excision wound healing model resulted in complete re-epithelialization compared with that in other groups [71]. Table 1 presents a list of anticancer drugs, lipids, polymers, cell lines, and applications of the developed liposomal system in the treatment of various cancers.

## 4. Dendrimers

**Figure 7:**
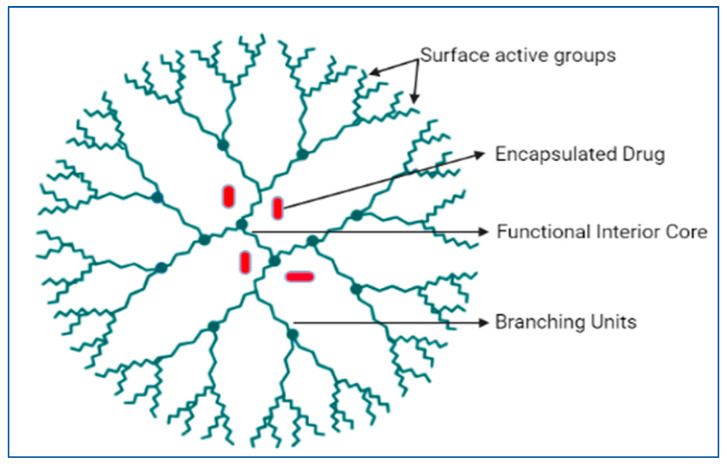
Diagrammatic representation of the dendrimer structure (created with BioRender.com, accessed on 25 August 2022).

Dendrimers are other starburst macromolecules that belong to the nanoscale drug delivery regime. The idea of dendrimers was first proposed by Flory, who was first synthesized by Voegtle [83] in 1978. Further, a group of researchers, Newkome, Yao, Baker, and Gupta [84], worked on dendrimer chemistry science. The word ‘Dendrimer’ is derived from the Greek words ‘dendron’ and ‘meros’, which mean tree and part, respectively [85]. In other words, they are monodispersed, three-dimensional (3D), and hyperbranched molecules. There are three parts, namely, the hyperbranched mantle, central core, and active surface or end groups [86]. Figure 7 shows a diagrammatic representation of the dendrimer structure. The inner core builds ‘cages and channels’ to ensure the easy creation of branching units, thereby accommodating bioactive principles. The branches endow the dendrimers with improved miscibility, high reactivity, and solubility.

Dendrimer surfaces can be complexed with bioactive ingredients with modifiable units [87]. Thus, dendrimers, by virtue of their structure, can entrap guest molecules, which are mostly lipophilic in the inner cavity. Owing to their monodispersity in size and shape, dendrimers are excellent nanocarriers through which diverse biomolecules can be entrapped either in the inner cavity or adsorbed to the surface or the terminal group of dendrimers. Additionally, dendrimers serve as ideal nanocarriers for increasing their solubility, providing controlled and sustained drug release, targeted delivery, and protection of active moieties from various therapeutic groups [88].

Bioactive phytochemical compounds such as curcumin, phenolic compounds, and carotenoids are hydrophobic, thus limiting their use in desired dosage forms [89]. The poor bioavailability of phytochemicals is due to their low aqueous solubility and poor intestinal permeability [90,91]. Dendrimers represent a unique class of molecules for solubilizing and delivering these active ingredients because of their polyvalency and nanoencapsulation properties [92,93]. Dendrimers offer protection of these bioactive ingredients from hostile conditions such as GI enzymatic degradation, variations in pH, and controlled release of the loaded drug.

Quercetin, a flavonoid phytochemical, is used as a dietary supplement. It possesses documented antioxidant [94], cardioprotective [95], anti-inflammatory [96], and anticancer [97] properties. Despite its numerous biological properties, its usage is limited by low drug insolubility and bioavailability. Madaan K et al. evaluated the potential of polyamidoamine (PAMAM) dendrimers as nanocarriers to improve the oral delivery of quercetin. They reported that in vitro drug release studies at 37 °C in phosphate buffer (pH 7.4) revealed a biphasic release pattern of quercetin with initial rapid release followed by a sustained release phase, hence proving their potential as carriers to resolve drug delivery-related issues [98]. In another study, Mekonnen et al. developed doxorubicin and resveratrol-loaded PAMAM dendrimer nanocomposite hydrogels to improve antitumor efficacy. Drug-loaded dendrimers entrapped in in situ hydrogels displayed significant synergistic antitumor activity against in vivo tumor-bearing mice [99].

Alginates are naturally occurring hydrophilic polymers obtained from brown seaweed. They are extensively studied and employed for various biomedical applications owing to their biocompatibility, safety, and low cost. The unique characteristic property of alginate is its ability to gelate in the presence of divalent cations, such as Ca^2+^, allowing it to be used in the formation of hydrogels [9].

The drug transport process in the hydrogel is pH-dependent, and further, the drugs encapsulated in alginate are not released at low gastric pH since they shrink. Once a compound arrives at the site of action in the intestine, the pH increases, subsequently resulting in rapid dissolution and drug release. It is a useful pharmaceutical material because of its multifaceted properties, such as nontoxicity, biocompatibility, biodegradability, nonantigenicity, and relatively low cost. All these features make them excellent drug delivery vehicles. Additionally, these materials can be integrated with other natural polymers, such as chitosan and pectin.

Numerous studies have shown that alginate-based nanoparticles can be used successfully for delivering antitumor drugs in cancer therapy [9]. An anticancer drug, the epirubicin nanogel, was prepared by crosslinking alginate and the G5 PAMAM dendrimer for breast cancer treatment. The nanoformula displayed sustained drug release under in vitro conditions [100]. Doxorubicin, a common chemotherapeutic agent, is encapsulated in the core of PAMAM dendrimers—modified mesoporous silica nanoparticles (MSNPs)—to yield doxorubicin-loaded nanoparticles for bladder cancer treatment. The resultant nanoparticles displayed the highest mucoadhesivity and controlled drug release, suggesting a prolonged bladder residence time of the drug [101].

In another study, Li et al. synthesized smart dual-responsive alginate hydrogels using sulfhydryl dendrimers as nanoblass-based drug delivery systems for cancer treatment. The hydrogel was prepared by the conjugation of a glutamic acid–cysteine dendrimer with alginate (Glu-Cys-SA) through click chemistry followed by cross-linking with PEG. In response to glutathione, a prominent component of the cellular antioxidative system, the doxorubicin-loaded dendrimer hydrogel displayed a controlled drug release pattern with an excellent release rate of 76% [102]. The hydrogels used as drug delivery systems in these studies offered benefits such as desirable therapeutic effects since hydrogels can reduce rapid drug release from dendrimers and have a high drug loading capacity, which helps ensure the sustainable and sequential release of drugs. These findings indicate that the sustained and controlled release of drugs can be attributed to the relatively slow degradation and high drug loading capacity of the hydrogel.

In addition to the use of dendrimers as carriers for phytochemicals, they are also widely utilized as ligands to increase the efficiency of drug encapsulation. Some examples of phytochemical-loaded dendrimers, as well as therapeutic drugs and their properties, are presented in Table 2.

## 5. Nanoshells

Nanoshells are a distinct class of nanoparticles composed of a dielectric silica core surrounded by a thin metal shell to treat cancer cells. They were first discovered by Naomi Hallas in 2003 [110]. Depending on its use, the shell can be a metal or oxide. This type of shell coating provides benefits such as the stabilization of colloidal dispersions, modification, and tailoring of particle properties and their inertness toward chemical reagents upon exposure to intense lasers [111].

Gold nanoshells are a special class of nanoshell particles. They possess special physical, chemical, and optical properties. Gold nanoshells are extremely useful in early cancer detection and treatment [112]. Nanoshells absorb infrared light, thereby transforming the energy of the light into heat energy. The resulting heat is quite strong for cancer cells to withstand. Due to their therapeutic photoinduction mechanisms, nanoshells are potential carriers of materials that support the elimination of bacteria and cancer cells. When using this method, there is no damage to nearby tissues [113]. By altering the thickness of the gold layer on the core silica particles, the optical properties and absorption of the gold nanoshells can be modified. The gold surface being biocompatible provides an added advantage to nanoshells for attaching various biomolecules [112]. Targeted drug delivery can be achieved by using nanoshells loaded with diverse targeting ligands, such as antibodies, peptides, proteins, and antibacterial and chemotherapeutic classes of drugs [114].

Since nanoshells possess large optical scattering properties, they can be employed as contrast agents in photonics imaging experiments. Among the diverse techniques of photonic imaging, reflectance confocal microscopy and optical coherence tomography are widely used since they aid in early cancer detection. The optical characteristics of nanoshells can be adjusted, making them suitable for cancer imaging as well as treatment. Through the selective accumulation of nanoshells, one can image tumors by utilizing the high permeability and retention features of cancer cells [112]. HER2 is a protein that stands for human epidermal growth factor receptor 2. Frequently, HER2 is overexpressed in cancer cells. Since nanoshells may specifically target and kill tumor cells without harming healthy cells, they are used in cancer therapy [112]. In addition to their application in cancer imaging and treatment, nanoshells are also used to synthesize a diverse range of coatings that provide long-lasting protection to various surfaces, such as stone, metal, wood, and glass. Such an application of the coating on these surfaces ensures protection of the surfaces without altering their original color, texture, etc. [111]. Gold nanorods (GNRs), as photothermal therapy (PTT), are widely used to cure cancer cells and tumor tissues because of their unique optical properties, biocompatibility, and easy surface modification. Joe et al. (2024) developed GNRs decorated with zinc oxide (ZnO) via Zn^2+^ hydrolysis in an alkaline environment to generate GNR@Zno nanocomposites. To improve the stability and therapeutic efficacy, the investigators functionalized GNR@Zno with CTPP (4-carboxybutyl) triphenylphosphonium bromide. The resulting all-in-one nanocomposite demonstrated effective cell ablation (95%) after 50 min of 780 nm NIR laser irradiation, suggesting that elevated temperatures can destroy cancer cells and induce reactive oxygen species (ROS), indicating that CTPP in the nanocomposite can specifically target the mitochondria [115].

## 6. Quantum Dots

Since quantum dots (QDs) display unique optical and electronic properties, they have been extensively researched as major probes for biomedical imaging under both in vitro and in vivo cancer conditions. They are composed of elements from groups II to VI or III to V, and they resemble semiconductor crystals. QDs emit fluorescence radiation after excitation with a light source. They help support the characterization of cells and lively creatures in fluorescent form [116]. QDs are semiconductor nanocrystals whose size typically ranges from 2 to 10 nm in diameter. Compared with conventional fluorescent dyes, QDs offer various advantages in spectroscopy, such as resistance to chemical degradation, strong and broad absorption patterns, high fluorescence intensities, high quantum yields, long lifetimes, high thermal stability, excellent resistance to photobleaching, and multifunctionality for different targeting ligands [117,118,119,120].

**Figure 8:**
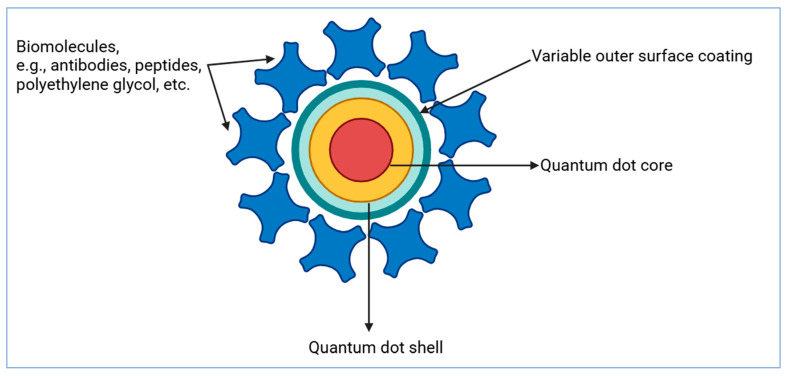
Schematic representation of quantum dot assembly (created with BioRender.com, accessed on 15 November 2024).

Small-molecule hydrophobic medications and imaging contrast agents are entrapped between the hydrophobic nanoparticle core and amphiphilic polymer coating, whereas hydrophilic therapeutics such as small interfering RNA (siRNA), antisense oligodeoxynucleotide (ODN), and various bioactive molecules, viz., peptides, antibodies, aptamers, polyethylene glycol, etc., can be immobilized onto the hydrophilic portion of the polymer coating through covalent or noncovalent bonding [121]. Figure 8 shows a schematic illustration of the QD assembly. Thus, a completely integrated nanostructure acts like a magic bullet that identifies, binds, or treats sick cells while also releasing observable signals through which one can perform real monitoring of nanostructures. Nanoparticle drug transporters offer many benefits, such as longer blood circulation, greater drug loading capacity, a controlled release pattern of medication, and the insertion of diverse targeting ligands on their surface, ensuring efficient and focused drug delivery [122]. Likewise, QDs are a novel approach in the assessment of drug administration because fluorescence may be used for tracking both the biodistribution and the intracellular take-up of a drug [123,124].

QD-based nanosystems offer several benefits over conventional imaging procedures. QD nanoparticles can be integrated with imaging labels or a mixture of labels for imaging studies, resulting in easy signal amplification and processing. Furthermore, higher binding affinity and specificity are attained through the attachment of several targeting ligands to the nanoparticles. Additionally, QD nanoparticles can be integrated with specific biomarkers to overcome biological barriers, resulting in enhanced drug targeting efficacy. Eventually, the fusion of imaging labels, various targeting ligands, therapeutic agents, and numerous other agents offers effective and controlled drug delivery in patients, thereby allowing real-time monitoring.

### 6.1. Detection of In Vitro Tumors

QDs play an important role in the early detection of tumors. They are used in fluorescence imaging owing to their photostable nature and high fluorescence emission [125]. They are widely employed in biomedical applications to investigate cell transport mechanisms, the movement of membrane transport proteins, in vivo diagnosis of hepatoma, intracellular delivery, and cell internalization by QDs [126]. Immunohistochemistry (IHC) is a powerful technique widely used for cancer diagnosis. An approach known as quantum dot immunohistochemistry (QD-IHC) is more accurate and precise at lower protein expression levels and allows for quantitative detection, which can aid in tailoring personalized treatment for patients [127,128]. Owing to good results in biomedical imaging, QD-based imaging has emerged as a new and promising technology for early cancer diagnosis [129,130].

Chung et al. developed fluorescent iron oxide and carbon dot-based nanoparticles through microwave irradiation and evaluated their utility in fluorescence imaging and treatment. The resultant quantum dots displayed good stability, and their fluorescence intensity and use in cancer cell lines revealed limited toxicity with swift and quantitative imaging [131]. Protein tyrosine kinase 7 (PTK 7), a membrane receptor tyrosine kinase, is often present in different types of cancer. Hence, the detection of this gene in the peripheral circulation could help in early cancer diagnosis. In one study, a selective and sensitive multicarbon dot and aptamer-based signal amplification ratiometric fluorescence probe was developed to detect PTK 7 in MCF-7 cells and human serum. The results indicated that the fluorescent sensor was successfully used for the detection of PTK7, which could eventually help in the diagnosis of cancer [132].

### 6.2. In Vitro Tumor Imaging

QDs belong to a new class of fluorescent probes that are used for a variety of biological and biomedical applications, including cellular imaging. Because of their multifaceted qualities, such as narrow emission spectra, good photostability, broad absorption spectra, and ability to generate all colors of QDs simultaneously via a single excitation light source, QDs act as excellent fluorescent probes and are preferred over conventional organic dyes [133].

Several investigations using QDs have documented in vitro fluorescence images of malignant cells from breast, ovarian, lung, melanoma, and adenocarcinoma cancers. Anti-type-I insulin-like growth factor receptor (IGF1R) is a molecule that is often overexpressed in breast cancer and is essential for the proliferation, survival, and metastasis of breast cancer cells. An IGF1R monoclonal antibody (AVE-1642) covalently coupled with QDs was developed by Zhang et al. to identify and quantify IGF1R levels. The resulting AVE-1642 QD complex slightly inhibited cell growth and reduced IGF1R levels in MCF-7 cells, indicating its potential for use as a therapeutic agent. Additionally, IGF1R quantum dots are suitable for focusing and in vitro tumor imaging of breast cancer cells [134].

QD-based staining in clinical tissue samples is another interesting in vitro biomedical application but is limited only to fixed section labeling. Since the HER2 receptor plays a vital role in the progression of aggressive forms of breast cancer, earlier identification of the HER2 receptor is important for both treatment and prognosis. However, conventional methods such as immunohistochemistry (IHC) still suffer from many issues, such as variable sensitivity, significant differences between laboratories, and subjective interpretation. Li et al. demonstrated HER2 expression in breast cancer through a QD-IHC system in an easy manner. The assessment of HER2 by QD-based probes was found to be accurate, superior, and sensitive over conventional methods [135].

### 6.3. In Vivo Tumor Imaging

In vivo fluorescence imaging involves the characterization and analysis of biomolecules in either the whole body of an experimental animal or an intact organism. In vivo tumor imaging presents different challenges, with the requirements of not only a specialized sensitive imaging agent but also the unique biological characteristics of tumorous tissue. Additionally, imaging studies in multicellular organisms are far more complex because of their greater size and thick and opaque tissue than cell studies in culture dishes or slides. Since tumorous cells are in contact with the constituents of the bloodstream, bioaffinity compounds can be used to actively target and distribute tumor-specific chemicals via their surface receptors. Since visible light cannot pass through biological tissues well, fluorescence imaging, a promising biomedical imaging modality widely used in animal models, has restricted use. However, most biological tissues consist of a near-infrared optical window that plays a vital role in deep-tissue optical imaging [136]. QDs are illumination nanoparticles due to their strong fluorescent signals and multifunctional nature, allowing a high degree of selectivity and sensitivity in cancer imaging. In vivo fluorescence imaging methods can be classified into four categories—the biodistribution of QDs in vivo, in vivo vascular imaging, in vivo tracking of QDs, and in vivo tumor imaging [129]. In vivo animal imaging, in vivo tumor focusing, and imaging are best investigated by examining the biodistribution and pharmacology of therapeutic QDs and performing in vivo vascular imaging.

#### 6.3.1. In Vivo Biodistribution

The systemic availability of QDs is influenced by a variety of parameters, including the type of targeting ligand, size, and shape. The size of the QDs and their tendency to bind to serum proteins determine the biodistribution of the QDs. Chitosan nanoparticles are linked covalently to chitosan, which has a high degree of acetylation, to develop fluorescent nanoparticle probes that are stable in the biological milieu needed for deep-tissue imaging [137]. Additionally, compared with PEG-QD nanoparticles, chitosan has an antioxidant effect and significantly enhances cellular uptake, thus allowing better tissue penetration and cell viability. Furthermore, in vivo studies revealed that the developed biocompatible chitosan-QD nanoparticles did not break down under harsh conditions, which further confirms that these nanoparticles can be utilized as versatile fluorescent nanoprobes. These encouraging achievements will further pave the way for long-term bioimaging applications and the development of highly sensitive multiplex in vitro assays.

#### 6.3.2. In Vivo Vascular Imaging

Angiogenesis is a process that involves both tumor cell proliferation and motility, both of which are essential. Tumor angiogenesis is significantly influenced by integrin v3. QDs have proven to be effective in vivo contrast agents for the cardiovascular system and the lymphatic system of mammals. Similarly, Smith et al. visualized blood vessel development, including angiogenesis, in the chorioallantoic membrane [138]. Various near-infrared and visible QDs have been utilized for imaging the blood vessels of chicken embryos, and the authors concluded that the QDs are more sensitive than conventional fluorescein conjugates. A20FMDV2, a peptide conjugated with copper indium selenide/zinc sulfide-based QDs, produces near-infrared (750 nm) to target αvβ6 integrin-rich head and neck squamous cell carcinoma (HNSCC) [139]. In vitro HNSCC models revealed that the QD-peptide bioconjugate nanoprobe was highly specific for αvβ6 integrin in two-dimensional (2D) monolayers and three-dimensional (3D) spheroids. With these positive results, scientists have concluded that the QD-A20 nanoprobe could be used as an effective nanoprobe for near-infrared (NIR) bioimaging and imaging-guided surgery.

#### 6.3.3. In Vivo Tracking of Cells

When QD-loaded cells are administered to living organisms, they emit fluorescence, thereby allowing the identification of the original cells and their offspring. QD-labeled cells were injected intravenously into mice to monitor the survival of QD-labeled cells in vivo for one week [140]. According to the fluorescence microscopy and flow cytometry data, the QD-loaded cells could remain in the peripheral blood for five days at a concentration of approximately 10%. In spleen sections, after two hours of injection, both EL-4 cells and QDs were detected. T lymphocytes were also observed and found to be amassed in the white pulp of the spleen. Even after 7 days, QD-labeled cells were identified in kidney, liver, and lung sections. Hence, it was concluded from these results that QDs could be used as potential bioimaging devices for the long-term tracing of in vivo target cells.

#### 6.3.4. In Vivo Tumor Based QDs

There are many problems related to in vivo tumor imaging, with the requirement of not only a particular and sensitive imaging agent but also the complex and multifaceted biological properties of tumor tissue. Cancerous tissue displays a unique feature called ‘enhanced permeability and retention’ (EPR), where nanoparticles ranging from ~10 to 100 nm are deposited. An angiogenic agent, vascular endothelial growth factor (VEGF), has an EPR effect, and an inefficient lymphatic drainage system in solid tumors results in the accumulation of macromolecules or nanoparticles. As a result, tumor-associated blood vessels develop abnormally with high permeability characterized by erratic architectures, leaky vessels, and pores. In addition, the presence of a poor lymphatic drainage system allows the extravasation of macromolecules and tumor components up to ~400 nm in size and their subsequent accumulation in the tumorous tissue [141,142]. This has paved the way for researchers to develop a wide range of nanobased systems for cancer treatment and imaging. QDs offer a high degree of specificity and sensitivity in tumor imaging because of their excellent fluorescent characteristics and multiplexing capabilities. In 2004, Gao et al. injected QDs bioconjugated with an antibody against prostate-specific membrane antigen (PSMA) via the intravenous route into mice to target and image human prostate cancer cells in vivo. Luminescent QD probes are bright and stable, display high tumor fluorescence, and possess a triblock copolymer structure, which ensures that they are suitable for coupling with therapeutic and diagnostic agents. Additionally, the polymer-encapsulated and bioconjugated QD probes were able to target tumor locations through passive and active mechanisms. Active targeting was found to be faster and more effective than passive targeting [143].

Recently, anticancer therapeutics conjugated with tumor-specific antibodies based on active tumor targeting have attracted increased interest in nanomedicine. This combinatorial approach will lead to increased therapeutic effects as well as a reduction in systemic toxicity. In diseases such as cancer with complex pathophysiology, QDs are currently used as powerful imaging agents in animal models. In 2005, Stroh et al. developed a combination of customized fluorescent semiconductor QDs and intravital microscopy to support tumor pathophysiology studies and the concurrent imaging and differentiation of tumor vessels from perivascular cells and the matrix. Using two-photon excitation, QDs were employed as fluorescent contrast agents for blood vessels, and simultaneously, the authors were able to capture images of perivascular cells from fluorescent protein expression and the extracellular matrix from autofluorescent collagen. The use of QDs results in a sharp and clear contrast for differentiating the components of the tumor owing to their high brightness, wavelength tunability, and reduced likelihood of extravasating into the tumor. Furthermore, they reported that quantum dot-loaded silicate beads of varying sizes displayed a size-dependent distribution of nanotherapeutics in tumors [144].

## 7. Intervention with Nanocarrier-Based Tumor Metabolism

Over the past few decades, enormous efforts have been undertaken to find effective cancer treatments because cancer is still a major illness that affects human health worldwide. The development of nanotechnology and biomaterials presents opportunities to safely and successfully integrate tumor metabolic intervention with cancer immunotherapies. The in vivo biodistribution and pharmacodynamic performance of macromolecular and small-molecule medications with immunomodulatory or tumor metabolic-rewriting capabilities can be enhanced by nanosized components that preferentially aggregate in solid tumor tissues. Owing to their advantages in multiple drug delivery, cell- and organelle-specific targeting, regulated drug release, and multimodal therapy, tumor metabolism-rewriting nanomedicines have recently become popular tactics for increasing anticancer immune responses. The development of multifunctional tumor metabolism-rewriting nanomedicines to induce antitumor immunity is summarized in this section. Particular attention is given to how these nanomedicines control the metabolism of glucose, amino acids, lipids, and nucleotides at the tumor site to increase innate or adaptive antitumor immunity.

Immune checkpoint modulators, such as ipilimumab, pembrolizumab, and durvalumab, which are approved by the U.S. Food and Drug Administration (FDA), offer long-term clinical benefits for specific cancer types, including melanoma, triple-negative breast cancer (TNBC), and NSCLC [145]. Nevertheless, the overall response rates of the majority of cancer patients to the aforementioned immune therapies are insufficient. This is due to the TMME, which suppresses the effectiveness of cancer immunotherapies by reducing a range of antitumor immune responses [146,147]. Aberrant metabolism of glucose, amino acids, lipids, and nucleotides results in intratumoral production and an accumulation of cytotoxic and immunosuppressive metabolites, leading to immune evasion by promoting the dysfunction and apoptosis of tumor-infiltrating immune cells (TIICs) [148,149]. Prostaglandin E2 (PGE2), generated by lipid metabolism, aids in tumor immune invasion by affecting the functions of T lymphocytes, myeloid-derived suppressor cells (MDSCs), and dendritic cells (DCs) [150]. Adenosine produced by the hydrolysis of adenosine triphosphate (ATP) triggers immunosuppressive adenosine receptor signaling, which in turn suppresses both T and B-cell activation, proliferation, and survival [151]. Interestingly, in addition to the production of immunosuppressive metabolites, metabolically active cancer cells can competitively reduce the intratumoral concentrations of glucose and different amino acids, such as glutamine, L-arginine, and lipids. All these are key nutrients that are essential for TIICs, including DCs and CD8^+^ effector T (Teff) cells [152,153]. Hence, in such nutrient-poor environments, the anticancer functions of TIICs may be severely compromised. In summary, there is hope for restoring antitumor immunity through rewriting or reprogramming the metabolically hostile TME [154,155].

Advancements in nanotechnology and biomaterials sciences have opened new avenues to integrate cancer immunotherapies with metabolic interventions for tumors in a safe and effective manner [156,157]. Nanosized compounds that accumulate in solid tumor tissues improve the pharmacodynamic performance and in vivo distribution of both large- and small-molecule drugs with immunomodulatory or tumor metabolic-rewriting capabilities. Since nanoparticles can penetrate the blood–brain barrier (BBB) and target specific cells or organelles, the drug-regulatory effects in the TMME may be enhanced [158,159]. In addition, nanomedicines can be tailored to produce controlled and spatiotemporal drug release in response to physical or biological stimuli in the TME, such as pH, enzymes, light, and glutathione (GSH) [160,161]. This strategy offers dual advantages—reduced side effects with enhanced therapeutic efficacy. Additionally, the integration of these tumor metabolism-rewriting nanomedicines with minimally invasive therapies such as chemodynamic therapy (CDT) and photodynamic therapy (PDT) can increase tumor immunogenicity and promote the formation of an inflammatory TME, leading to synergistic immunotherapeutic effects [162,163]. When combined, the multifunctional properties of nanomedicines provide several advantages in modulating the metabolism of cancer and boosting antitumor immunity in a synergistic manner.

### 7.1. Intervention in Glucose Metabolism

Glucose is an important source of carbon and energy for tumor cells; hence, it plays a central role in the metabolism of tumors. Even in the presence of moderate amounts of oxygen, cancer cells metabolize and convert glucose to lactate via a process known as aerobic glycolysis or the Warburg effect [164]. Although this process results in the production of less ATP than oxidative phosphorylation, cancer cells can still generate glycolytic intermediates for faster cell division [165]. Thus, targeting glucose metabolism may be an effective way to eliminate cancer cells selectively. Importantly, the increased uptake of glucose by tumor cells results in an intratumoral glucose shortage, which suppresses T-cell activation and differentiation as well as the release of proinflammatory cytokines from Teff cells [166,167]. Moreover, higher levels of lactate produced during aerobic glycolysis result in acidosis in the tumor microenvironment, which in turn could promote immunosuppression and aggressive proliferation [168]. Thus, inhibiting tumor glucose metabolism and removing immunosuppressive lactate from the TME serve as two useful therapeutic strategies for enhancing anticancer immune responses. Here, we provide a summary of the advancements in nanomedicines that can increase antitumor immunity by altering glucose metabolism in tumor cells, tumor-associated macrophages (TAMs), and natural killer (NK) cells.

#### 7.1.1. Rewriting Tumor Cell Glycolysis

A literature review suggested that lactate generated from tumor glycolysis can stimulate the polarization of tolerogenic M2-type TAMs, which in turn inhibits the activation and proliferation of TIICs such as Teff cells, DCs, and NK cells [169,170]. Hence, the inhibition of glucose metabolism can impair lactate-mediated immune suppression and promote anticancer immunotherapy. In this context, Li et al. developed a pH-sensitive nanosystem (SK/siR-NPs) composed of the glycolysis inhibitor shikonin (SK) with PD-L1 small interfering RNA (siRNA) loaded in a folic acid (FA)-modified polymeric network [171]. The shikonin released from the nanosystem was able to decrease lactate production through downregulation of a key glycolytic enzyme—pyruvate kinase-M2 (PKM2)—thereby promoting repolarization of TAMs from the M2 subtype to the M1 subtype to eliminate the immunosuppressive environment. Furthermore, the effective inhibition of PD-L1 resulted in enhanced T-cell function against tumors.

In addition to other mechanisms, activated TIICs, such as Teff cells, TAMs, NKs, and DCs, use glycolysis to exert their tumoricidal effects [172,173]. Thus, indiscriminate suppression of cellular glycolysis at the tumor location may compromise the anticancer effects of TIICs. Therefore, it is imperative to develop treatment approaches that selectively target and inhibit tumor cell glycolysis and spare TIICs. Pyruvate dehydrogenase kinase 1 (PDK1), a highly expressed enzyme found in mitochondria, plays a vital role in tumor glycolysis and is a potential target for inhibiting glycolysis in cancer cells [174]. Dichloroacetate (DCA), a PDK1 inhibitor, has low therapeutic efficacy because of its reduced cellular uptake and poor mitochondrial localization. To address these limitations, a triphenylphosphonium (TPP) cation-modified nanomedicine (T-Mito-DCA-NP) system was developed to transport DCA to mitochondria—targets resulting in a reprogrammed immunosuppressive TME [175]. The resulting system specifically downregulates PDK1 in mitochondrial tumor cells, thus reducing the rates of glycolysis and lactate production. Additionally, the coadministration of T-Mito-DCA-NP and anti-PD-1 in in vivo murine colon and breast cancer models enhanced Teff cell infiltration and decreased immune checkpoint protein expression.

In the case of glycolysis inhibition, cancer cells utilize nicotinamide adenine dinucleotide (NADH) to produce ATP as an alternative energy source. Gliomas are characterized by the overexpression of two metabolic enzymes, namely, ALDH1L1 and PKM2. For these reasons, Zhao et al. fabricated blood–brain barrier (BBB)-penetrating albumin/lactoferrin hybrid biomimetic nanoparticles loaded with shikonin (PKM2 inhibitor) and disulfiram (ALDH1L1 inhibitor) at a fixed dose ratio for antiglioma therapy [176]. Disulfiram interferes with tumor energy metabolism by blocking the supply of ATP through an alternative pathway, in which ALDH1L1 plays a crucial role. This nanoplatform displayed good BBB penetration owing to the ability of albumin to bind to the albumin-binding proteins (e.g., SPARC) present in cells of the BBB. Additionally, the increased cellular uptake of nanomedicine was attributed to the binding of lactoferrin to LRP-1, which is expressed in tumor cells. After entering tumor cells, shikonin, via PKM2 inhibition, downregulates glycolysis and decreases the production of lactate. Notably, the reduced levels of lactate in tumor tissues promoted the differentiation and proliferation of CD8^+^ Teff cells, thus increasing antitumor immunity. Disulfiram inhibited ALDH1L1 to downregulate the NADH-ATP energy metabolic pathway. Through this dual inhibition strategy, both glycolysis and NADH-ATP metabolism can be simultaneously suppressed, eventually resulting in a decrease in ATP generation in tumor cells.

#### 7.1.2. Rewriting Intratumoral Lactate Levels

**Figure 9:**
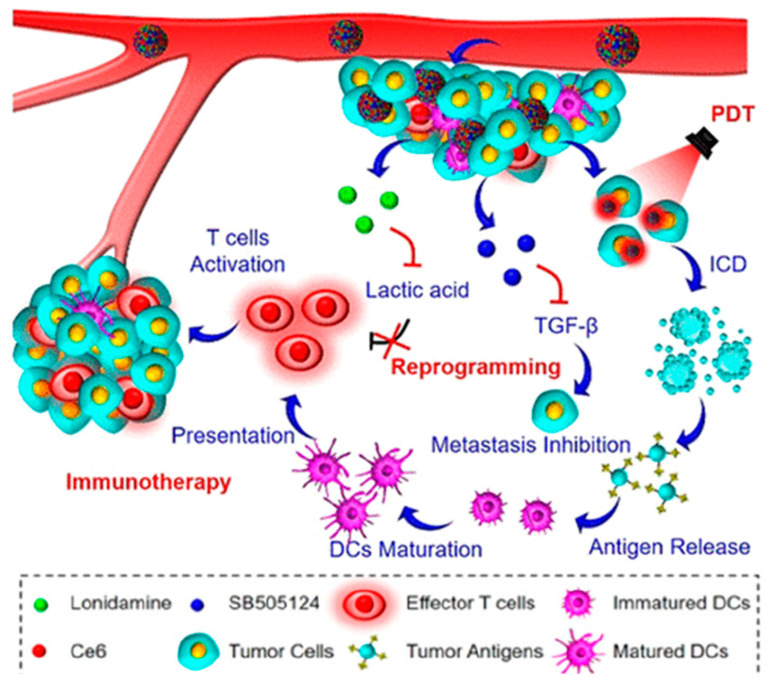
Nanomedicines used for rewriting intratumoral lactate levels. Figure illustrating TerBio’s ability to self-assemble and its roles in tumor metabolism reprogramming and PDT-sensitive immunotherapy. This figure was adapted/reproduced from [177] with permission from the American Chemical Society, copyright 2017.

Numerous techniques aided by nanotechnology have been developed to lower the levels of lactate in the TME. These include inhibiting lactate efflux and promoting lactate intake. To reduce intratumoral lacate levels and enhance photodynamic therapy (PDT) against colorectal cancer, a ternary bioregulator delivery system (TerBio) was developed [177]. The TerBio system was developed using chlorin e6 (Ce6), the transforming growth factor (TGF)-β receptor-I inhibitor SB505124 (SB), and the lactate efflux inhibitor lonidamine (LND) at a suitable ratio without the need for extra excipients (Figure 9). TerBiocomplex-mediated PDT inhibited tumor growth and induced immunogenic cell death (ICD), which triggered strong anticancer immune responses. Additionally, the decrease in lactate efflux induced by LND and SB-induced TGF-β downregulation jointly improved antitumor immunity by mitigating the immunosuppressive TMME (Figure 9).

Furthermore, in vivo experiments in CT26 tumor-bearing mouse models revealed that the TerBio system was able to suppress primary and distant tumor growth and decrease tumor metastasis.

Intratumoral lactate can be eliminated with the help of the catalytic enzyme lactate oxidase (LOX), which converts lactate into hydrogen peroxide (H_2_O_2_) and pyruvate [178]. In this context, He et al. developed a novel lactate treatment plant, i.e., a nanofactory (PLNP^Cu^) composed of LOX and Cu^2+^ in a cationic-based PEI nanoassembly, for synergistic chemodynamic therapy (CDT) and metabolic therapy [179]. With the help of the PEI component, which acts as a reservoir of LOX, the designed system can actively trap lactate and increase lactate degradation 2-fold. Notably, H_2_O_2,_ the breakdown product of lactate degradation, can be converted to generate strongly oxidizing hydroxy radicals (OHs) with antitumor properties in the presence of copper ions, thereby inducing ICD. Thus, the downregulation of lactate-mediated immunosuppressive TMME and reactive ROS-induced ICD synergistically resulted in a potent antitumor immune response in mice bearing 4T1 breast cancer tumors.

#### 7.1.3. Rewriting Glycolysis in Tumor-Associated Macrophages (TAMs)

**Figure 10:**
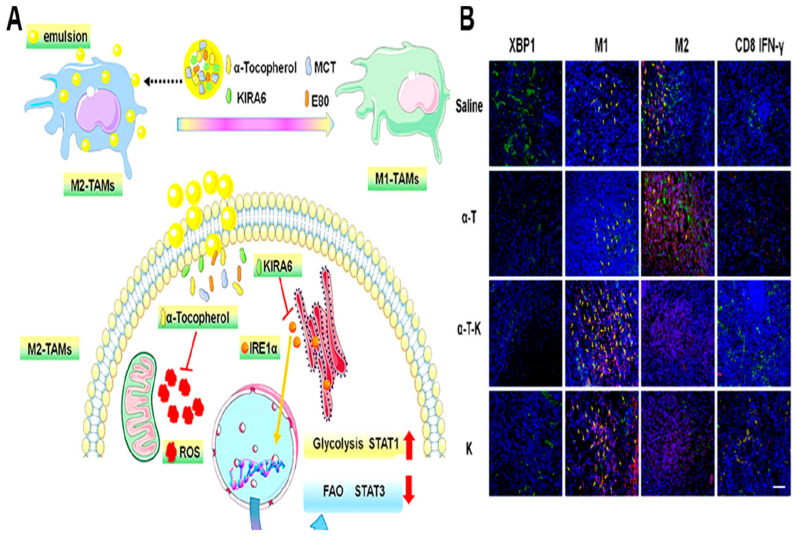
Nanomedicines used for rewriting glycolysis in TAMs. (**A**) Diagram illustrating how α-T-K promotes the polarization of M2-like TAMs toward M1-like phenotypes by simultaneously inhibiting oxidative stress and endoplasmic reticulum stress. (**B**) The distributions of XBP1 (green), M1 (F4/80^+^TNF-α^+^, yellow arrow), M2 (F4/80^+^CD206^+^, yellow arrow), and cytotoxic CD8^+^ T (CD8 green, IFN-γ red) cells in tumor tissues are depicted in representative immunofluorescence images. This figure was adapted/reproduced from [186] with permission from the American Chemical Society, copyright 2021.

The polarization characteristics of tumor-associated macrophages (TAMs) are determined by their metabolic profiles [180]. Aerobic glycolysis is the energy source used for proinflammatory M1 TAMs, whereas M2 TAMs that promote tumor growth heavily rely on OXPHOS to carry out protumor-associated activities [181]. In the hypoxic TME, cancer cells prevent TAMs from aggregating, which in turn reduces glucose competition and promotes metastatic growth [182]. Hence, one possible strategy to increase antitumor immunity is to control the metabolic phenotype of TAMs. Recent studies have indicated that M2—TAMs can shift to M1—like phenotypes under normal levels of oxygen due to the suppression of phosphatidylinositol 3-kinase gamma (PI3Kγ) and the activation of nuclear factor kappa-B (NF-κB) [183,184]. Since M2-like TAMs are often present in hypoxic tumors, the polarization of M1-like TAMs is hindered [185]. To overcome the above limitations, Jiang et al. developed a nanoemulsion composed of α-tocopherol (α-T-K, an oxidative stress inhibitor), and KIRA6, an endoplasmic reticulum (ER) stress inhibitor [186] (Figure 10A). Under hypoxic conditions, the nanosystem enhanced antitumor immunotherapy by driving TAM polarization from the M2 phenotype to the M1 phenotype. α-T-K-mediated suppression of both oxidative stress and ER stress leads to increased glycolysis and decreased fatty acid oxidation (FAO) in cancer cells.

This alteration in cellular metabolism resulted in reduced numbers of M2-like TAMs with increased infiltration of cytotoxic CD8^+^ Teff cells in tumor tissues (Figure 10B). Additionally, in vivo experiments revealed that α-T-K was not only able to delay breast tumor growth in a 4TI-induced breast cancer model but also to enhance the therapeutic effects of PD-1 antibody-based immunotherapy in Lewis lung carcinoma (LLC) model mice. In another study, Gu et al. developed a nanosystem using a ferroptosis-inducing agent (RSL3) loaded with an iron-based metal–organic framework to simultaneously regulate both inflammatory and metabolic functions, leading to the safe and strong activation of TAMs [187]. Degradation of this nanoplatform in acidic lysosomes results in the release of iron, thereby promoting lipid peroxidation and impairing the functions of M2-like TAMs. This combination synergistically induced mitochondrial dysfunction in M2-like TAMs, thereby causing a metabolic switch from mitochondrial oxidative phosphorylation to glycolysis, thus promoting the polarization shift toward the M1-like phenotype. Consequently, the nanoplatform promoted proinflammatory M1 signaling-associated pathways while inhibiting anti-inflammatory signaling pathways. Furthermore, this nanomedicine exhibited strong antitumor effects on TAMs, leading to phagocytic death and metastasis suppression.

#### 7.1.4. Rewriting Glycolysis in Natural Killer (NK) Cells

**Figure 11:**
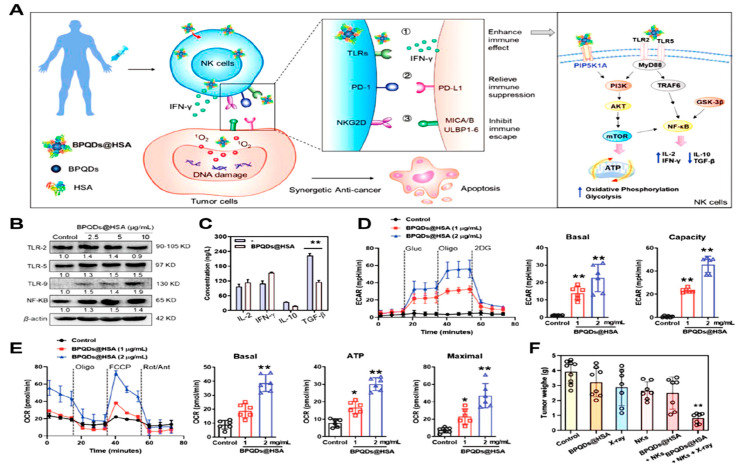
Nanomedicines used for rewriting glycolysis in NK cells. (**A**) Diagram illustrating how BPQDs@HSA enhances the tumoricidal properties of NK cells generated from patients. (**B**) Western blot analysis of EGFR, mTOR, and GSK-3β expression levels in NK cells following BPQD@HSA therapy. (**C**) Impact of BPQDs@HSA on the production of cytokines by NK cells (*n* = 3). (**D**) The OCRs of NK cells treated with BPQDs@HSA for a full day were examined following the addition of FCCP, rotenone, oligo, and antimycin (Rot/Ant). (**E**) Following the progressive addition of glucose (Gluc), oligomycin (Oligo), and 2-deoxy-d-glucose (2DG), the glycolytic capacity and basal glycolysis of NK cells treated with BPQDs@HSA for 24 h were assessed via the ECAR. (**F**) Tumor weight changes in a HepG-2 xenograft model following various treatments (*n* = 10). * *p* < 0.05, ** *p* < 0.01. This figure was adapted/reproduced from ref. [189] with permission from the Wiley Online Library, copyright 2023.

Natural killer (NK) cells are important immune cells that can recognize and eliminate cancer cells. The tumoricidal effect of NK cells is due to the release of cytolytic granules and cytotoxic cytokines, which are regulated by a complex network of activating and inhibiting receptors. However, the modification of metabolic pathways impairs the functions of NK cells in the TME [188]. He et al. synthesized highly efficacious and biocompatible black phosphorous quantum dots encapsulated with a human serum albumin (BPQDs@HSA) immunosensitizer to promote NK cell activity against tumor cells [189] (Figure 11A). BPQD@HSA therapy of NK cells increased the expression of the Toll-like receptors (TLRs) TLR-5, TLR-9, and NF-Κb (Figure 11B). Additionally, NF-κB signaling activation significantly increased NK cell secretion of cytokines such as IFN-γ and decreased the release of immunosuppressive proteins such as TGF-β and IL-10 (Figure 11C).

BPQDs@HSA function as phosphate group donors when they bind with the protein phosphatidylinositol 4-phosphate 5-kinase type-1 gamma (PIP5K1A). This, in turn, activated the downstream PI3K-AkT and mTOR signaling pathways, thereby reprogramming the cellular metabolism of glycolysis (Figure 11D) and improving OXPHOS (Figure 11E), which together aid in preserving NK cell viability and immunity. Both the BPQDs@HSA- and X-ray irradiation-treated HepG-2 xenograft models displayed greater penetration of NK cells in tumor tissues, resulting in a significant tumor inhibition rate of up to 81.1% (Figure 11F).

### 7.2. Amino Acid Metabolism Intervention

Amino acid metabolism plays a vital role in cancer development and progression. Tumor cells exhibit modified amino acid metabolism, which in turn supports rapid growth and proliferation. In addition to promoting biosynthesis and energy metabolism, amino acid metabolism also plays a crucial role in regulating antitumor immunity [153].

T-cell activation and differentiation are limited by a lack of intratumoral amino acids, which results from the high metabolic rates of tumor cells [190,191]. Additionally, the key enzymes involved in amino acid metabolism, viz., 2,3-dioxygenase (IDO) and tryptophan 2,3-dioxygenase (TDO), increase intratumoral production and the accumulation of immunosuppressive metabolites, e.g., Kyn, thereby affecting the functions of T cells [153]. Hence, researchers can target amino acid metabolism in tumors to create an immunostimulatory TME, which can enhance tumor immunotherapy. Here, we discuss nanomedicines that synergistically enhance antitumor immunity by regulating tryptophan and L-arginine metabolism in the TME.

#### 7.2.1. Nanomedicines Used for Rewriting Tryptophan Metabolism in Cancer Cells

**Figure 12:**
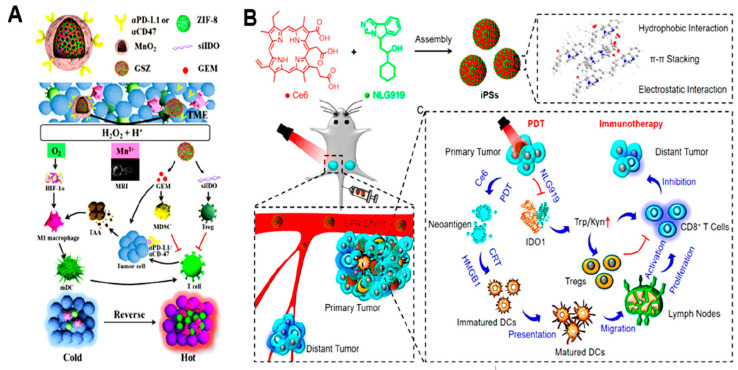
Nanomedicines used for rewriting tryptophan metabolism in cancer cells. (**A**) Nanomodulator (GSZMP) preparation and suggested mechanism to improve ICB-based tumor immunotherapy. This figure was adapted/reproduced from ref. [198] with permission from Elsevier, copyright 2020. (**B**) Diagram illustrating the process of creating carrier-free nanoassemblies (iPSs) for tumor immunotherapy sensitive to PDT. This figure was adapted/reproduced from ref. [199] with permission from the American Chemical Society, copyright 2020.

Higher levels of tryptophan-degrading enzymes, e.g., IDO and TDO, in tumor cells and other cells, e.g., endothelial cells, MDSCs, and TAMs, accelerate the consumption of tryptophan [192]. Interestingly, IDO promotes the accumulation of Kyn, a clinically validated immunosuppressor that helps in tumor immune escape [193]. Kyn increases the recruitment of immunosuppressive Tregs and MDSCs while hindering tumor infiltration and proliferation and the activation of Teff cells [194,195]. Hence, the inhibition of IDO is a potential strategy by which tumor immunosuppression can be mitigated [196]. Several IDO inhibitors, along with immune checkpoint inhibitors (ICIs), have displayed positive results in clinical trials [197]. In the context of nanomedicine-assisted therapy, Chen et al. constructed a nanocage (GSZMP) for codelivering small interfering RNA targeting indoleamine 2,3-dioxygenase-1 (siIDO) and gemcitabine (GEM) into MnO_2_-mineralized ZIF-8 nanoparticles (zinc 2-methyl imidazole), followed by surface decoration with anti-PD-L1 [198] (Figure 12A). Tumors enriched with H^+^ and H_2_O_2_ induced the disintegration of the mineralized MnO_2_ shell, resulting in O_2_ generation and the repolarization of M2-like TAMs to M1-like phenotypes. Following internalization by tumor cells, the ZIF-8-based nanocages disintegrated in acidic endosomes, thereby enabling the quick escape of siIDO and GEM via a proton sponge mechanism. GEM-induced ICD supported the maturation of DCs by facilitating the release of tumor neoantigens (Figure 12A).

On the other hand, siIDO-mediated IDO inhibition significantly reduced Kyn production, resulting in enhanced tumor infiltration of Teff cells and the reversal of the ‘cold’ immune tumor milieu to a ‘hot’ state, thereby potentiating immune checkpoint blockade (ICB)-based treatment for ‘cold’ malignancies. In another study, Zhao et al. developed a carrier-free nanoassembly consisting of the self-delivery photoimmune stimulator (iPS) chlorine e6 (Ce6) as a photosensitizer and an immunomodulator (NLG919) for photodynamic sensitized tumor immunotherapy [199] (Figure 12B). Without the need for extra excipients, both Ce6 and NLG919 self-assembled into nanoparticles via the combined effects of hydrophobic interactions, π-π stacking, and electrostatic interactions. The resulting self-assembled iPSs displayed improved solubility, stability, biocompatibility, excellent drug loading rates, and minimal systemic toxicity. ROS produced by iPSs under light irradiation destroy tumors and produce neoantigens to trigger an ICD and antitumor immune response. Additionally, NLG919 was able to reverse the immunosuppressive TME via IDO inhibition and tumor infiltration of Treg cells, thereby inhibiting both primary and distant tumors through the photodynamic sensitized immunotherapy of iPSs.

#### 7.2.2. Nanomedicines Used for Rewriting L-Arginine Metabolism in Cancer Cells

**Figure 13:**
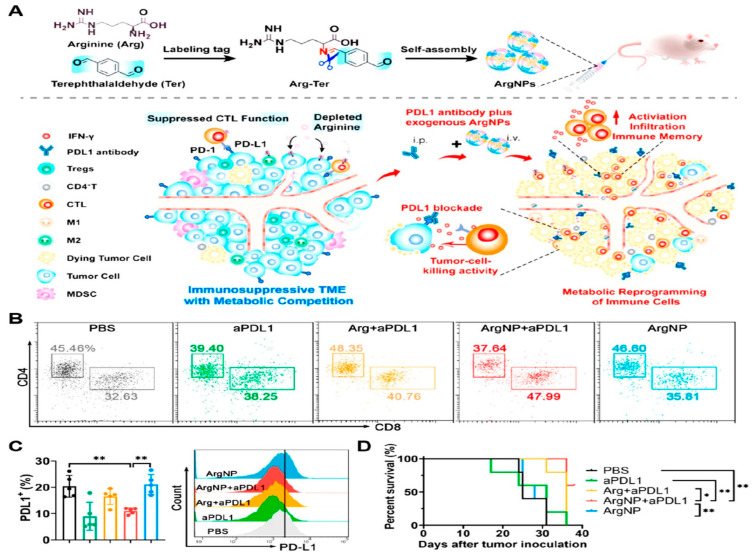
Nanomedicine used for rewriting L-arginine metabolism. (**A**) Diagram illustrating how ArgNPs are made and how they work to improve ICB-based immunotherapy through metabolic intervention in tumors. (**B**) Representative flow cytometry analysis of the proportions of CD4^+^ and CD8^+^ Teff cells in tumor tissues after different treatments (gated on CD3^+^ T cells). (**C**) PD-L1 expression on tumor cells subjected to different treatments was analyzed via flow cytometry. (**D**) Survival curves of 4T1 tumor-bearing mice after different treatments. The data are expressed as the means ± SDs (* *p* < 0.05, ** *p* < 0.01, *n* = 5). This figure was adapted/reproduced from ref. [205] with permission from Elsevier, copyright 2023.

The availability of arginine (arg) in the TME is vital since it supports antitumor immune responses, particularly the differentiation and activation of Teff cells [200,201]. According to earlier research, in vitro depletion of arg hinders the functions of tumoricidal T cells, including the production of IFN-γ, CD3ζ chain expression, and apoptosis [202,203]. Hence, arg supplementation can be used as a possible tactic to sustain T-cell-based immunity against tumors. However, the direct intratumoral administration of highly hydrophilic arg poses several issues, such as enzymatic degradation and quick outward diffusion of the compound within the TME. Consequently, these factors significantly lower the arg concentration, thereby impairing therapeutic efficacy at the tumor site [204]. For this purpose, a pH-responsive nanoarginine (ArgNP) based on dynamic tag-mediated self-assembly that can improve ICB therapy metabolically was developed [205] (Figure 13). To construct nanoassemblies with high drug loading capacity, terephthalaldehyde (Ter) was added to arg by forming acid-sensitive imine bonds, thereby increasing the hydrophobicity of arg (Figure 13A). At pH 5.0, the acid-labile imine bond is cleaved, resulting in ArgNPs disassembling and releasing arg.

The flow cytometry results revealed that ArgNP and anti-PD-L1 combined promoted the infiltration of CD8^+^ Teff cells and increased the ratio of CD8^+^/CD4^+^ T cells in tumors (Figure 13B). Notably, the combination of ArgNP and anti-PD-L1 treatment resulted in reduced PD-L1 expression in tumor cells (Figure 13C). Additionally, in vivo studies demonstrated that ArgNPs plus an anti-PD-L1 antibody significantly inhibited tumor growth and prolonged the survival period in 4T1 tumor-bearing mice (Figure 13D).

## 8. RNA-Based Cancer Therapy

Therapeutic RNAs, viz., messenger RNAs (mRNAs), small interfering RNAs (siRNAs), small noncoding RNAs (microRNAs), and single guide RNAs (sgRNAs), possess superior tumor-suppressing capabilities since they can either upregulate the expression of target tumor-suppressor genes or suppress the expression of target oncogenes [206,207]. mRNA technology offers a myriad of benefits, such as easy design and modification of RNA sequences, rapid production and scalability, and delivery of therapeutic proteins directly to cancer cells. Unlike chemotherapy, which has side effects, RNA-based therapy is minimally toxic and does not result in drug resistance in tumors. However, RNA-based therapy is less effective because the human immune system has natural defense mechanisms (e.g., exonucleases and RNAses) that catalyze the breakdown of exogenous RNAs, thus hampering the utilization of therapeutic RNAs for in vivo cancer treatment [208]. The abovementioned limitations can be addressed with the aid of nanoparticle-mediated delivery platforms since nanoparticles improve the chemical stability and pharmacokinetic profile of encapsulated RNAs to a great extent [209]. Nonviral nanoparticles not only offer protection to therapeutic RNAs from enzymatic degradation but also specifically accumulate in tumor tissues via the enhanced permeability and retention (EPR) effect, thus reducing off-target toxicity [210]. NPs can be designed so that they are able to release therapeutic RNAs in response to environmental stimuli such as target cell endosomes and acidic tumor microenvironments [211]. Additionally, nanoparticles can be tagged or labeled with cell-specific targeting ligands and moieties to enhance their cell and nucleus penetration [212]. Eventually, nanoparticle-mediated RNA therapies have the potential to improve the accuracy of cancer treatment, target specific signaling pathways, and increase the effectiveness of existing chemotherapies.

### Nanoparticles Used for mRNA Delivery

The restoration of tumor-suppressing proteins is an effective therapeutic strategy for cancer treatment [213]. Phosphate and tensin homolog (PTEN), a tumor-suppressor gene, is lost or mutated in approximately half of patients with metastatic castrate-resistant prostate cancer (mCRPC). In patients with mCRPC, PTEN inhibits the P13-AKT signaling pathway, thereby promoting cancer cell survival, proliferation, and migration [214]. For this purpose, Islam et al. developed self-assembling polymer poly(lactic-*co*-glycolic acid PLGA)-lipid (GO-C14) hybrid nanoparticles coated with a polyethylene glycol (PEG) shell for the effective delivery of therapeutic PTEN mRNA into PTEN-null prostate cancer cells. The systemic administration of this hybrid delivery system in prostate cancer-bearing mice resulted in significant tumor inhibition, increased apoptosis, and blockade of the P13–AKT signaling pathway [215]. The same research group developed another self-assembling lipid–polymer hybrid nanoparticle delivery system consisting of therapeutic p53 mRNA into p53 null hepatocellular carcinoma (HCC) and NSCLC cells. A total of 36% of HCC patients and 68% of non-small cell lung cancer (NSCLC) patients have a defective p53 tumor-suppressor gene [216]. p53 promotes the transcription of proapoptotic proteins in the nucleus, such as BCL-2-associated X (BAX) and the p53-upregulated modulator of apoptosis (PUMA). Furthermore, prosurvival autophagy, which leads to multidrug resistance (MDR) in cancer, is inhibited by p53 in the cytoplasm.

## 9. Conclusions

The primary goal of the application of nanotechnology in cancer is to provide an effective treatment for this disease. Eminent cancer therapy techniques, such as chemotherapy and radiotherapy, have several limitations. With the use of nanocarriers such as liposomes, dendrimers, nanovectors, nanoshells, and quantum dots for drug delivery, nanotechnology has increased the possibility of treating cancer, and chemotherapy-associated limitations have been overcome to a certain extent. These nanoformulations, as well as polymeric nanoparticles, increase drug solubility, uptake, and bioavailability, thus reducing side effects. The use of cancer nanotechnology has increased expectations for treatment. This review highlights various trending nanocarriers, biopolymers, their potential as drug delivery systems, and nanotechnology-assisted metabolic intervention in tumors to enhance antitumor immune responses. We summarize the latest developments in the engineering of nanomedicines that can synergistically enhance antitumor immunity by ‘reprogramming or rewriting’ the metabolism of glucose and amino acids, and this has become a popular and successful strategy. Despite this, major challenges must be addressed with respect to the TMME, such as the development of alternative metabolic pathways in tumor cells, the acquisition of resistance, the development of nanomedicines that can target selective metabolic activities, and the limited accumulation and penetration of nanomedicines, thereby reducing their therapeutic potential. To overcome these limitations, efforts are needed to develop nanomedicines that can regulate both tumor metabolism and the complex TME simultaneously. Additionally, we discuss the therapeutic potential of mRNAs generated via nanodelivery platforms that upregulate the expression of tumor-suppressor genes and downregulate the expression of oncogenes. Preclinical studies have indicated that nanoparticles can efficiently and effectively deliver RNA for use in cancer therapy. Prior to their clinical use, nanoparticles must be thoroughly assessed to determine their biodistribution, toxicity, and clearance parameters. Additionally, their successful clinical translation depends upon industrial-scale production via good manufacturing practices (GMPs) and batch-to-batch reproducibility. All these considerations are highly important for developing personalized nanotherapy for patients. Furthermore, extensive efforts are required in research on the development of new specialized nanoparticles through which an increasing number of drugs can be targeted. With the invention of new conjugation techniques, nanotechnology will experience a continually expanding list of applications. Although significant attention is being given to nanotechnology, equal attention is needed in laboratories that produce nanomedicines so that they can record themselves in clinical trials. Additionally, the results of these medicines in clinical trials are overwhelming, with reduced side effects and their ability to alter the dose of the drug. Future directions in cancer nanotechnology include the development of self-regulating systems.

## Figures and Tables

**Table 1:** Lists of lipids, polymers, cell lines, and anticancer medications and how the established liposomal system is used to treat different types of cancer.

Drug/Carrier	Polymers/Lipids/Adjuvants	Animal Model/Cell Lines	Application/ Indications	Ref
Gemcitabine-doxorubicin-MPLA liposomes	DSPC and PEG-2000, Cholesterol, monophosphoryl lipid A (MPLA)	orthotopic 4T1 murine breast cancer tumor model	Breast cancer	Wu D et al. [72]
PEG-folate-coated liposomes containing PTX (SpHL-FT-PTX)	DOPE SPC DSPE-PEG2000 DSPE-PEG2000-folate	Human breast adenocarcinoma cell lines (MCF-7 and MDA-MB-231	Breast cancer	Barbosa et al. [73]
Thymoquinone-loaded liposomes	DPPC Triton X-100	Breast cancer cell lines (MCF-7 and T47D)	Breast cancer	Odeh et al. [74]
Doxorubicin-loaded magnetic liposomes	HSPC, DSPE, and cholesterol	colorectal cancer (CT-26 cells)	Colorectal cancer	Hardiansyah et al. [75]
Doxorubicin and erlotinib loaded Transferrin-cell penetrating peptide-penetratin liposomes	DOTAP, DOPE, cholesterol, DSPE-PEG (2000)-Pen	orthotopic brain tumor mice model	Glioblastoma (Brain tumor)	Lakkadwala et al. [76]
Celastrol liposomes	DOPC, cholesterol, DSPEmPEG2000, DSPC,	VCaP human prostate cancer cells	Prostate cancer	Wolfram et al. [77]
Resveratrol liposomes	Tripeptide lipid CDO and sucrose laurate L126	human breast cancer MCF-7 cells	Breast cancer	Zhao et al. [78]
DTX-PTGS Liposomes	DPPC, cholesterol and either DSPE–mPEG2000 or TPGS	C6 glioma cells (To assess cellular uptake and cytotoxicity)	Brain cancer	Muthu et al. [79]
Multifunctional targeting epirubicin liposomes	EPC, cholesterol, DSPE-PEG2000, DSPE-PEG2000-OCT, and honokiol	C57BL/6 mice injected with LLT cells	non-small cell lung cancer	Song et al. [80]
Transferrin-functionalized liposomes loaded with Docetaxel	SPC: Chol: DSPE-PEG (2000)-Mal	prostate cancer cell line PC-3 and normal human prostate cell line PNT2	Prostate cancer	Fernandes et al. [81]
Coloaded paclitaxel/rapamycin liposomes	SPC, cholesterol, DSPE-PEG, DPPC, and 1,2-DSPC	4T1 cell line; mice	Breast cancer	Eloy et al. [82]

**Table 2:** Examples of phytochemical-loaded dendrimers.

Active Ingredient	Dendrimer Type	Size of Complex (nm)	Zeta Potential (mV)	Nanoencapsulation Efficiency (%)	Ref.
Doxorubicin	Polyamidoamine	432	−20.7	52	[103]
2-methoxyoestradiol (2-ME)	Polyamidoamine	-	2.41 ± 4.25	-	[104]
Berberine	Polyamidoamine	210.7 ± 9.98 nm	30.3 ± 0.69	37.9	[105]
Reseveratrol	Poly ethyleneglycol-poly lactic acid	153.37 ± 28.2	−9.6 ± 0.4	78.2 ± 4.8	[106]
Gemcitabine	Polyamidoamine	~200 nm	−8.9 to −21.9	22.07–40.05	[107]
Cisplatin	Polyamidoamine	20–40 nm	-	21.10 ± 0.453	[108]
Curcumin	Poly(lactic acid)	102.8 ± 1.2	−16.5 ± 2.1	18.5 ± 1.3	[109]

## Data Availability

The dataset supporting the conclusions of this article is available from the corresponding author upon request.
